# Visually evoked potentials may be abnormal in COVID-19 patients if the infection is complicated by cerebral disease

**DOI:** 10.1590/1806-9282.20240412

**Published:** 2024-07-19

**Authors:** Josef Finsterer, Carla Alexandra Scorza, Fulvio Alexandre Scorza

**Affiliations:** 1 Neurology and Neurophysiology Center – Vienna, Austria.; 2 Universidade Federal de São Paulo, Paulista School of Medicine – São Paulo (SP), Brazil.

Dear Editor,

We read with interest Balduz and Fidancı's article about a prospective, single-centre, case-control study on the difference between pattern reversal visually evoked potentials (VEPs) and flash VEPs in 44 patients with recent SARS-CoV-2 infection compared to 40 controls^1^. Pattern reversal VEPs did not differ between COVID-19 patients and controls, but right-side P2 latency in flash VEPs was prolonged in COVID-19 patients^1^. A total of 13 patients had increased P2 latency^1^. The study is impressive, but several points require discussion.

The first point is that the conclusions drawn are unsupported^1^. The number of patients was too low and the mono-centric design was inappropriate to draw such conclusions. Since only a single parameter was abnormal (P2 latency on the right side), it is quite unlikely that VEPs are generally abnormal in COVID-19 patients in the absence of severe neurological complications.

The second point is that the cause of right P2 prolongation has not been reported^1^. We should know the results of clinical neurological examination, cerebral imaging, electroencephalography (EEG), and cerebrospinal fluid (CSF) analysis in all patients with P2 prolongation. Has a neurological cause been identified that could explain the finding?

The third point is that the cause of the headache was not specified in almost two-thirds of patients^1^. We should know whether these patients had primary or secondary headache, history of headache, or experienced headache after SARS-CoV-2 infection. The most common causes of secondary headache reported in association with SARS-CoV-2 infection include arterial hypertension, meningitis/encephalitis, intracerebral bleeding, subarachnoid bleeding, venous sinus thrombosis, reversible cerebral vasoconstriction syndrome, dissection, sleep disorders, or desiccosis, which must be thoroughly excluded.

The fourth point is that it remained unclear why P2 was prolonged on the right side but not on the left side. Assuming that P2 prolongation was due to SARS-CoV-2 infection, one would expect bilateral rather than unilateral prolongation. How do the authors explain this unusual finding?

The fifth point is that differential diagnoses, such as new-onset multiple sclerosis, new-onset neuromyelitis optica spectrum disorder (NMO-SD), MOG-associated disorder (MOG-AD), acute disseminated encephalomyelitis (ADEM), posterior reversible encephalopathy syndrome (PRES), acute, hemorrhagic necrotising encephalopathy (AHNE), acute, hemorrhagic leukoencephalitis (AHLE), and acute, necrotizing encephalopathy (ANE), were not adequately excluded. Since these conditions can be associated with unilateral P100 or P2 prolongation^2,3^, it is imperative to have them off the table before attributing them to SARS-CoV-2 infection.

The sixth point is that the origin of reference limits was not described. We should know whether reference limits for VEP parameters were generated by the authors themselves or were taken from the literature or a book.

In summary, the excellent study has limitations that should be addressed before drawing final conclusions. Clarifying the weaknesses would strengthen the conclusions and improve the study. VEPs in COVID-19 patients may only be abnormal when the visual pathway is compromised by SARS-CoV-2 infection, which is the case with encephalitis, stroke, bleeding, or immunological disease triggered by COVID-19.
